# The ‘Pants-Sign’: A Predictor for Falling in People with Parkinson’s Disease?

**DOI:** 10.3233/JPD-230353

**Published:** 2023-12-19

**Authors:** Jamie A.F. Jansen, Anouk Tosserams, Vivian G.M. Weerdesteyn, Bastiaan R. Bloem, Jorik Nonnekes

**Affiliations:** a Radboud University Medical Center, Donders Institute for Brain, Cognition and Behavior, Department of Rehabilitation, Center of Expertise for Parkinson & Movement Disorders, Nijmegen, The Netherlands; b Radboud University Medical Center, Donders Institute for Brain, Cognition and Behavior, Department of Neurology, Center of Expertise for Parkinson & Movement Disorders, Nijmegen, The Netherlands; c Department of Rehabilitation, Sint Maartenskliniek, Ubbergen, The Netherlands

**Keywords:** Parkinson’s disease, falls, postural instability, risk factors, balance

## Abstract

**Background:**

A history of falls is the most established predictor of future falls in people with Parkinson’s disease (PD). However, predicting a first fall remains challenging.

**Objective:**

To assess whether experiencing difficulties putting on pants while standing is a viable predictor of future falling, and specifically a first fall, in persons with PD. We define this ‘Pants-sign’ as people who resort to putting on their pants only while seated.

**Methods:**

264 persons with PD were included. Information on the Pants-sign, history of falls, disease severity (MDS-UPDRS part III), freezing of gait (N-FOGQ > 0), cognitive function (MoCA), self-reported disability (Schwab & England scale), health-related quality of life (SF-12), Timed-Up-and-Go, and one-legged stance were determined at baseline and after one-year follow-up. The association between the Pants-sign and future falling was examined by univariate logistic regression analysis. A multivariate step-wise logistic regression with forward selection was employed to identify the strongest associations in the entire cohort and a sub-cohort of people without falls in the year prior to baseline.

**Results:**

The Pants-sign was univariably associated with a future fall (OR = 2.406, 95% CI [1.313–4.409], *p* = 0.004]), but was not an independent predictor in the multivariate logistic regression; predictors were higher MDS-UPDRS part III scores (OR = 1.088, 95% CI [1.056–1.121], *p* < 0.001] and history of falls (OR = 5.696, 95% CI [2.650–12.243], *p*≤0.001]. For the sub-cohort of people without falls in the previous year (*n* = 189), the Pants-sign was not associated with future falls.

**Conclusions:**

The Pants-sign is simple to assess and is associated with future falling in PD but is not an independent predictor.

## INTRODUCTION

People with Parkinson’s disease (PD) commonly experience falls [1, 2]. Overall, they are nine times more likely to fall compared to healthy elderly persons [3]. Falls and fall-related injuries have a significant impact on quality of life, as it may lead to fear of falling, reduced mobility, and loss of independence [4, 5]. Furthermore, experiencing a fall may induce a complex vicious circle in which fear of falling, reduced balance confidence, avoidance behavior, and negative physical conditioning ultimately lead to more falls and fall-related injuries in the future [6]. Importantly, these negative downstream consequences can be tackled through timely interventions [7].

In order to facilitate the appropriate application of interventions to mitigate fall risks, it is important that healthcare professionals can identify persons with PD at risk of future falling. Earlier studies have thus far identified several risk factors for future falls in people with PD. The most important predictor of a future fall is a history of prior falls (i.e., two or more falls in the previous year) [8, 9], but a longer disease duration, a greater disease severity, presence of freezing of gait, reduced gait speed, and presence of cognitive impairment are also associated [9–12]. Despite these known risk factors, predicting future falls remains challenging, particularly in patients without a history of falls [13, 14]. Hence, there is a need for additional tools to reliably identify people with PD at risk of future falls. Ideally, such assessments should be readily applicable and not take much time in daily clinical practice.

Fall risks in people without a history of falls (i.e., identifying the very first set of falls) might potentially be deduced from simple questions regarding a patient’s ability to perform general activities of daily living that involve balance performance. An example of such an activity is putting on pants while standing. This notion emerged during everyday (but uncontrolled) observations in our own clinical practice, where persons with PD who also manifested other signs of posture instability frequently indicated that they were no longer able to put on their pants while standing. Here, we decided to take this observation to the test. Specifically, we investigated whether having stopped putting on pants while standing (we will refer to this as the ‘Pants-sign’) could be a predictor of future falling in people with PD. Subsequently, we specifically assessed whether the presence of the Pants-sign could be a viable predictor of a first fall in persons with PD without a history of falls.

## METHODS

### Study population

Data for this study were obtained from a large ongoing longitudinal cohort study (the “Personalized Parkinson Project”) that aims “*to identify new biomarkers that can assist in predicting differences in prognosis and treatment response between patients*” [15]. People with early PD (< 5 years disease duration) participated in the Personalized Parkinson Project. Participants underwent three comprehensive annual assessments at the Radboud university medical center in Nijmegen, the Netherlands. These assessments comprised home-based self-reported measures and on-site clinimetrics. During the on-site assessments, the Pants-sign was identified by asking: ‘*Did you stop putting on your pants (or skirt) while standing, because this isn’t easy anymore?*’. Importantly, this question was introduced during the course of the Personalized Parkinson Project and was not included from the start. Therefore, not all persons with PD answered this question during all assessments. To obtain the largest study population, we used available data from the second assessment as the baseline and used the assessment one-year after for follow-up. The study protocol was approved by the local ethics committee (Commissie Mensgebonden Onderzoek (CMO) Region Arnhem-Nijmegen (reference number 2016-2934; NL59694.091.17)). All participants provided written informed consent before participation.

Information on the number of falls in the past year and the presence of freezing of gait (defined by a score of > 0 on the New Freezing of Gait Questionnaire [NFOG-Q]) [16] were obtained from home-based questionnaires. Motor assessment consisted of the MDS-UPDRS part III [17], the Timed Up-and-Go test [18] and the one-legged stance test (ability to stand on one leg for 10 s) [19]. Cognitive assessment comprised the Montreal Cognitive Assessment (MoCA) [20]. Self-reported disability was measured using the Schwab and England scale [21], and health-related quality of life scores were assessed using the Short Form Health Survey (SF-12) [22]. Participants were included when all data were available.

### Statistical analyses

Statistical analysis was performed using IBM SPSS 27 (SPSS, Inc, Chicago, IL). To identify differences between persons with and without the Pants-sign, data were tested for normality using the Shapiro-Wilk test, and then compared using either an independent samples T-test, or a non-parametric independent Kruskal-Wallis test. The variables sex, presence of a fall in the past year, more than one fall in the past year and presence of freezing of gait were compared using a 2×2 chi-square test of homogeneity.

The association between the Pants-sign and a future fall, i.e., a fall in the year after baseline, was identified using a 2-step exploratory approach. A binomial logistic regression analysis was used to test all possible associated variables univariately. Then, associated variables were entered in a step-wise binomial logistic regression analysis with forward selection (likelihood ratio method) to determine the best model fit. First, these steps were executed for the total cohort to determine the validity of the Pants-sign as a predictor for future falling in general. Subsequently, these steps were performed for a sub-cohort of people without a fall in the previous year to determine the predictive value of the Pants-sign regarding a first fall. Alpha was set at *p* < 0.05.

## RESULTS

In total, 264 participants were included in the final analysis (Table 1). Twenty-two percent of the cohort (*n* = 58) reported the Pants-sign at baseline (i.e., these people had stopped putting on their pants while standing). At baseline, people with the Pants-sign had more often experienced at least one fall in the preceding year (43%) compared to people without the Pants-sign (24%, *p* = 0.005). Additionally, people reporting the Pants-sign had a longer disease duration, had a higher disease severity, were more often freezers, had a lower MoCA score, had greater self-reported disability, had a lower health-related quality of life score (SF-12) and performed worse on the retropulsion test, the Timed Up-and-Go test and the one-legged stance test compared to people without the Pants-sign.

**Table 1:** Study population characteristics

	Total Cohort	Pants-sign	No Pants-sign	*p* ^1^
	*n* = 264	*n* = 58	*n* = 206
Age (y)	62.4±8.5	62.1±7.7	62.6±8.7	0.644
Men (*n*, %)	151 (57%)	30 (52%)	121 (59%)	0.340
Time since diagnosis (y)	3.2±1.3	3.6±1.3	3.2±1.3	0.031^*^
Estimated time since onset of motor symptoms (y)	5.4±3.3	6.2±3.9	5.2±3.0	0.024^*^
Fallen in the past year	75 (28%)	25 (43%)	50 (24%)	0.005^*^
More than one fall in the past year	34 (13%)	18 (31%)	16 (8%)	< 0.001^*^
Presence of freezing of gait^2^ (*n*, %)	46 (17%)	20 (34%)	26 (13%)	< 0.001^*^
MDS-UPDRS part III score (median, [0–136])
ON	30 [7–62]	30.5 [11–62]	30 [7–58]	0.295
OFF	36 [10–69]	38.5 [17–69]	35 [10–64]	0.026^*^
Hoehn and Yahr stage (median, [1–5])	2 [1–3]	2 [2–3]	2 [1–3]	0.931
Retropulsion test (median, [0–4])
ON	1 [0–3]	1 [0–3]	1 [0–3]	0.002^*^
OFF	1 [0–3]	1 [0–3]	1 [0–3]	< 0.001^*^
Timed Up-and-Go (seconds)
ON	6.76±1.67	7.48±2.25	6.56±1.41	0.008^*^
OFF	7.23±2.30	8.25±2.79	6.94±2.06	< 0.001^*^
Abnormal one-legged stance test^3^ (*n*, %)
ON	25 (9%)	13 (22%)	12 (6%)	< 0.001^*^
OFF	33 (12%)	19 (33%)	14 (7%)	< 0.001^*^
Self-reported disability^4^ (%)	90±8.1	88±7.6	92±8.1	< 0.001^*^
MoCA score (median, [0–30])	27 [17–31]	26 [18–30]	27 [17–31]	0.047^*^
SF-12 score^5^
Physical Composite Score	49.3±9.9	43.4±10.6	50.1±9.1	< 0.001^*^
Mental Composite Score	35.0±5.7	33.6±5.9	35.4±5.6	0.030^*^

Values are represented as mean±SD, unless otherwise specified. ^1^Comparison between the Pants-sign cohort versus the no Pants-sign cohort. Data was tested for normality using the Shapiro-Wilk test, and then compared using either an independent samples T-test, non-parametric independent Kruskal-Wallis test. Sex, Fallen in the past year, more than one fall in the past year and presence of freezing of gait were compared using a 2×2 chi-square test of homogeneity. ^2^Defined by a score of > 0 on the NFOG-Q. ^3^Cut-off value of 10 seconds. ^4^Defined by Schwab & England test for self-reported disability. ^5^Short Form Health Survey.

Variables associated with a future fall are presented in Table 2. When considering the entire cohort, the Pants-sign was univariately associated with a future fall (OR = 2.406, 95% CI [1.313–4.409], *p* = 0.004]). The multivariate analysis revealed that a history of recurrent falls (OR = 5.696, 95% CI [2.650–12.243], *p*≤0.001]) and a higher MDS-UPDRS part III score (OR = 1.088, 95% CI [1.056–1.121], *p* < 0.001]) were independent predictors of future falls (Table 2). The Pants-sign did not significantly contribute to the model.

**Table 2:** Baseline characteristics associated with a fall in the year after baseline: all participants

Total cohort (*n* = 264)	Odds ratio	95% CI	*p*	*R*^2 =^0.240
**More than one fall in the past year**	**5.696**	**2.650–12.243**	**< 0.001**
**MDS-UPDRS part III *ON***	**1.088**	**1.056–1.121**	**< 0.001**
Presence of Pants-sign	2.406	1.313–4.409	0.004
Age	1.050	1.015–1.086	0.004
Years since diagnosis	1.459	1.188–1.793	< 0.001
Fallen the past year	2.313	1.314–4.072	0.004
Presence of freezing of gait	2.122	1.101–4.088	0.025
SF-12: Physical composite score^1^	0.974	0.949–1.000	0.050
Self-reported disability^2^	0.923	0.889–0.958	< 0.001
MoCA score	0.861	0.773–0.959	0.007
MDS-UPDRS part III *OFF*	1.076	1.046–1.106	< 0.001
Timed Up-and-Go time *ON*	1.397	1.180–1.653	< 0.001
Timed Up-and-Go time *OFF*	1.334	1.164–1.529	< 0.001
Abnormal retropulsion score (score > 0)**ON*^3^*	2.264	1.099–4.663	0.027
Abnormal retropulsion score (score > 0)*OFF^3^*	2.462	1.088–5.571	0.031

Variables that were included in the analysis of the cohort that included all participants by means of forward selection are presented in bold, and determined the R^2^ per model. CI, confidence interval ^1^Short Form Health Survey. ^2^Defined by Schwab & England. ^3^Based on score of the retropulsion test in the MDS-UPDRS part III item 3.12.

When considering the sub-cohort of people without a reported fall in the previous year, the Pants-sign was not associated with a prospective fall. For this sub-cohort, a higher MDS-UPDRS part III score (OR = 1.082, 95% CI [1.040–1.126], *p* < 0.001]) was the only significant predictor of a first fall (Table 3).

**Table 3:** Baseline characteristics associated with a fall in the year after baseline: subcohort consisting of people that did not report a fall in the year preceding baseline

Non-fallers at baseline (*n* = 189)	Odds ratio	95% CI	*p*	*R*^2^ = 0.132
**MDS-UPDRS part III *ON***	**1.082**	**1.040–1.126**	**< 0.001**
Age	1.067	1.023–1.112	0.002
Years since diagnosis	1.294	1.008–1.662	0.043
MoCA score	0.866	0.764–0.981	0.024
MDS-UPDRS part III *OFF*	1.061	1.024–1.100	0.001
Timed Up-and-Go time *ON*	1.291	1.026–1.625	0.029
Timed Up-and-Go time *OFF*	1.261	1.052–1.511	0.012
Abnormal retropulsion score (score > 1) *ON^1^*	4.000	1.434–11.155	0.008
Abnormal retropulsion score (score > 1)*OFF^1^*	3.407	1.224–9.489	0.019

Variables that were included in the analysis of the cohort that consisted of people that did not report a fall at baseline by means of forward selection are presented in bold, and determined the R2 per model. CI, confidence interval. ^1^Based on score of the retropulsion test in the MDS-UPDRS item 3.12, score > 0.

## DISCUSSION

This study explored whether having stopped putting on pants while standing (the ‘Pants-sign’) could be a predictor of future falling in people with PD. Our results indicate that the Pants-sign is indeed associated with future falling in people with PD, but has inadequate independent predictive value in the presence of a history of prior falls and disease severity (MDS-UPDRS part III). The Pants-sign failed to predict a first fall in people with PD without a fall history.

Our finding that disease severity and a history of falls are important predictors for a future fall is in line with the literature [3, 8, 10, 11, 23, 24]. We extend this earlier work by showing that the Pants-sign has some added value in predicting a future fall, albeit not independently from other known predictors, and only among those who already experienced prior falls. In this specific population, it might still be useful to consider asking the question about putting on pants, as it is very simple quick to ascertain, particularly in a busy everyday clinical practice. Asking about prior falls also remains important, but this can be challenging due to recall bias—it is critical to always involve the caregiver during the interview for that reason. Our work confirms that ascertaining disease severity is also important, but there will be very few clinicians who complete a complete MDS-UPDRS motor examination in a busy clinic. Moreover, there is no specific cut-off value for the MDS-UPDRS score that invariably predicts the future fall. Another problem is that the MDS-UPDRS motor score has a U-shaped correlation to the risk of falls, such that the risk of falling appear to taper off again with further advancing disease severity, presumably because people with the greatest instability become immobile and therefore no longer sustain falls [8]. So we feel that asking about the Pants-sign may have some value, although with limitations.

One particular limitation is the fact the Pants-sign had no predictive value in the falls-naïve sub-cohort, where the need for early prediction is highest. Five previous studies reported on predictors of future falls either in fall-naïve PD patients [14], patients without a fall in the previous six months [25, 26], or patients without a fall in the previous year [27, 28]. A cohort study involving 121 persons with PD identified that the combination of gait speed slower than 1.13 m/s, a single-leg support time lower then 659 ms, and a H&Y stage of III increased the risk of a first fall in the coming 36 months by eight times compared to patients without these features [14]. In contrast to our study, a higher disease severity score (as measured by the MDS-UPDRS part III) was not the strongest predictor. A reason could be the difference in study populations since the latter study included newly-diagnosed participants within 4 months of diagnosis, and our study included patients within five years of diagnosis. It is surprising that the latter cohort study [14] included 15 newly diagnosed patients with a H&Y stage of III, a finding which is uncommon at the time of diagnosis. It is possible that some of these individuals actually had a form of atypical parkinsonism. In another study that included 130 persons with PD that did not report a fall in the past year, a multivariable analysis showed that self-reported disability (Schwab and England scale) was the strongest associated predictor for a future fall [27]. Disease severity (UPDRS part III) was associated with future falls in the univariable analysis but was not a predictor in the multivariate analysis. We also included the Schwab and England scale as a measure for self-reported disability, but only found a univariable association with a future fall in the entire cohort, but not in the cohort that did not fall in the previous year. This is in line with a cohort study involving 101 PD patients [28], which reported that the UPDRS part II score (as a measure of self-reported disability) was associated with a future fall, but not in a sub-cohort of 59 PD patients that did not fall in the previous year. Despite differences in the outcomes between these studies, markers of disease severity and self-reported disability seem to be relevant predictors for falls in fall-naïve PD persons.

It is interesting consider further why the Pants-sign was correlated to future falls in those with prior falls, but not within the fall-naïve subgroup. One possibility is that the prior falls triggered a fear of falling, which subsequently motivated people to become more cautious and start putting on their pants while seated. Certainly, our current findings suggest that the very first fall was not preceded by such subjective feelings of instability, or at the least that these patients had not yet begun to take precautionary measures.

A limitation might be the room for interpretation of the question itself, since it did not specify the timeline of having stopped to put on pants while standing. Another limitation is that we did not include the presence of co-morbidities into our analyses. Importantly, in addition to Parkinson’s disease, co-morbidities can also contribute to the change in behavior leading to the adoption of sitting while putting pants on. One particular comorbidity that can impact the ability to put on pants while standing is polyneuropathy; its incidence has reported to be increased in persons with PD versus controls [29]. We did not objective the presence of polyneuropathy in our cohort but recommend this to be included in future studies. Furthermore, we included people that were diagnosed with PD recently (on average 3 years ago, with a maximum disease duration of five years), and our findings may not generalize to the entire PD population. It is therefore worthwhile to study the Pants-sign in more severely affected populations, or those with a form of atypical parkinsonism. However, due to this inclusion criterium, there was a substantial proportion of fall-naïve PD participants in the present study, which favored the investigation of the association between the Pants-sign and a first fall.

Taken together, this study confirms several established predictors of falls, including prior falls and greater disease severity, and also suggests that the Pants-sign might be a simple measure to help predict future falls in busy clinics, when applied to a population that has already sustained the first falls. We did not find a new effective measure to predict a first fall in people with PD, but we hope that our findings help to shape the agenda for further studies to investigate better predictors that allow for accurate fall risk assessment in fall-naïve patients.

## Data Availability

The data supporting the findings of this study are available on request from the corresponding author.
